# Engineering cancer cell membrane-camouflaged metal complex for efficient targeting therapy of breast cancer

**DOI:** 10.1186/s12951-022-01593-5

**Published:** 2022-09-05

**Authors:** Xiaoying Li, Yanzi Yu, Qi Chen, Jiabao Lin, Xueqiong Zhu, Xiaoting Liu, Lizhen He, Tianfeng Chen, Weiling He

**Affiliations:** 1 Department of Neurology and Stroke Center, The First Affiliated Hospital, Department of Chemistry, Jinan University, Guangzhou, 510632 China; 2 Department of Obstetrics and Gynecology, the Second Affiliated Hospital of Wenzhou Medical University, Wenzhou, China; 3 Department of Gastrointestinal Surgery, The First Affiliated Hospital, Center for Precision Medicine, Sun Yat-sen University, Guangzhou, Guangdong 510080 China

**Keywords:** Metal complex, Cancer cell membranes, Camouflage, High biocompatibility, Cancer targeting therapy

## Abstract

**Background:**

Cancer cell membrane-camouflaged nanotechnology for metal complex can enhance its biocompatibility and extend the effective circulation time in body. The ruthenium polypyridyl complex (RuPOP) has extensive antitumor activity, but it still has disadvantages such as poor biocompatibility, lack of targeting, and being easily metabolized by the organism. Cancer cell membranes retain a large number of surface antigens and tumor adhesion molecules CD47, which can be used to camouflage the metal complex and give it tumor homing ability and high biocompatibility.

**Results:**

Therefore, this study provides an electrostatic adsorption method, which uses the electrostatic interaction of positive and negative charges between RuPOP and cell membranes to construct a cancer cell membrane-camouflaged nano-platform (RuPOP@CM). Interestingly, RuPOP@CM maintains the expression of surface antigens and tumor adhesion molecules, which can inhibit the phagocytosis of macrophage, reduce the clearance rate of RuPOP, and increase effective circulation time, thus enhancing the accumulation in tumor sites. Besides, RuPOP@CM can enhance the activity of cellular immune response and promote the production of inflammatory cytokines including TNF-α, IL-12 and IL-6, which is of great significance in treatment of tumor. On the other hand, RuPOP@MCM can produce intracellular ROS overproduction, thereby accelerating the apoptosis and cell cycle arrest of tumor cells to play an excellent antitumor effect in vitro and in vivo.

**Conclusion:**

In brief, engineering cancer cell membrane-camouflaged metal complex is a potential strategy to improve its biocompatibility, biological safety and antitumor effects.

**Supplementary Information:**

The online version contains supplementary material available at 10.1186/s12951-022-01593-5.

## Introduction

 The outstanding characteristics of complexes, including their unique molecular structures, the ligand exchange, redox and catalytic offer these compounds the opportunity to react with biomolecules, which makes them as bioactive therapeutic compounds with promising applications in tumor therapy [1, 2]. Varieties of compounds have been approved for therapeutic and imaging purposes in the clinical. Up to now, they have a wide range of applications in cancer treatment, which has been considered as promising agents, including chemotherapy, photodynamic therapy and imaging guided treatment [3]. Metal complexes represented by cisplatin exhibited the broad spectrum of antitumor activity, and still act as the first-line drugs in the clinical tumor treatment. Advent of cisplatin chemotherapeutic drug and its powerful anticancer effect have aroused great interest in metal anticancer drugs among scientists. Till now, many kinds of metal-containing compounds have been investigated for their applications in therapy and diagnostics in cancer treatment, such as ruthenium complexes, [4, 5] iridium complexes, platinum complexes, and gold complexes etc [6–10]. For instance, Liang et al. have reported a tailored multifunctional anticancer system for ruthenium-based photosensitizers, which can be excited by the near-infrared two-photon light source to remodel tumor microenvironments and enhance the combined cancer therapeutic effect [4]. *Wang et al* found that iridium (III) complex (Ir1) can generate damage-associated molecular patterns (DAMPs) and increase endoplasmic reticulum stress and reactive oxygen species, eventually result in long-acting anti-tumor immunity in lung cancer cells [9]. Although many metal complexes have been proven excellent in cancer therapy and entered clinical trials, they also suffer from similar shortcomings, such as inevitable toxicity against normal cells, low biocompatibility and solubility under physiological conditions, which impedes their development in further clinical application [11]. Therefore, it is important to find effective approaches to replace these shortcomings of metal complexes.

With rapid development of nanotechnology, nano-engineering of metal complexes has made great progress in the diagnosis and treatment of malignant tumors to overcome their shortcomings [12, 13]. Scientists have made great efforts in modification of metal complexes by various approaches such as metal oxide nanomaterials with enzyme mimicking activities, polymer, liposome, micelles and inorganic nanoparticles decoration, as well as in combination with other anticancer drugs [14, 15]. Although liposome offered high biocompatibility and simple formulation, polymer-based nanocarriers exhibited versatility for hydrophilic and hydrophobic drugs, they also exhibited unsatisfying toxicity and stability issues [16]. Besides, it is very difficult for metal-based nanodrugs to identify and enter tumor areas, and they often lose their efficient effects under the attack of the immune system [17–19]. Therefore, it is very important to construct a biomimetic nanoplatform for metal complex, which can avoid the attack of the immune system and decrease its clearance. Instrinkly, the cell membrane encapsulating nanotechnology has gradually attracted attention for their maintained cells surface biochemical characteristics, such as antigens and cell adhesion molecules, which can be used as a bioinspired nanotechnology to prevent nano-drug clearance, enhance the biocompatibility and blood circulation time in vivo [20–23]. Red cells were used to camouflage nanoparticles, which further confirmed that compared to polyethylene glycol-modified particles, the half-life of nanoparticles wrapped in red blood cell membranes in mice is longer, with a half-life of up to 40 h in circulation [24]. In addition, on surface of tumor cell membranes there exists a special antigen, which can specifically identify homologous tumors and prolong the retention and homing ability in tumor site [25–27]. On the one hand, compared to other membrane, nanoparticles wrapped in tumor cell membranes have an active targeting, which can rely on the infiltration of capillaries in the tumor microenvironment to the tumor site, further actively aggregate to tumor lesions through the same recognition mechanism [28]. On the other hand, tumor cell membrane-camouflaged nanoparticles can activate the immune system and improve the clearance of pathogen by utilizing the characteristic proteins of tumor cell membranes [29]. For instance, Shen et al. have constructed a cancer cell membrane camouflaged iridium complexes functionalized black-titanium nanoparticles of Ir-B-TiO_2_@CCM, and found that compared with unencapsulated nanoparticles, the homologous targeting and immune escape properties of cancer cell membranes promote the selective accumulation of Ir-B-TiO_2_@CCM to homologous tumor cells, while avoiding the immune rejection of macrophages, and improving the safety of tumor accumulation and treatment [30]. Besides, Cancer cells possess lots of excellent properties, including immune escape and homologous targeting abilities etc. During the phase of metastasis, homotypic cancer cell aggregation is important for establishing secondary lesions in distant organs. It is also reported that the aggregation process is based on surface adhesion molecules on cancer cell membranes. Cytotoxic drugs have been demonstrated that some drugs induce immunogenicity by expressing tumor-specific antigens and MHC-I molecules on the surface of cancer cells [31]. Collectively, rational design of bioinspired nanomaterials with cell membrane camouflaging technology for metal complex is a new strategy to overcome their shortcomings and enhance the cancer treatment [32–34].

**Scheme 1:**
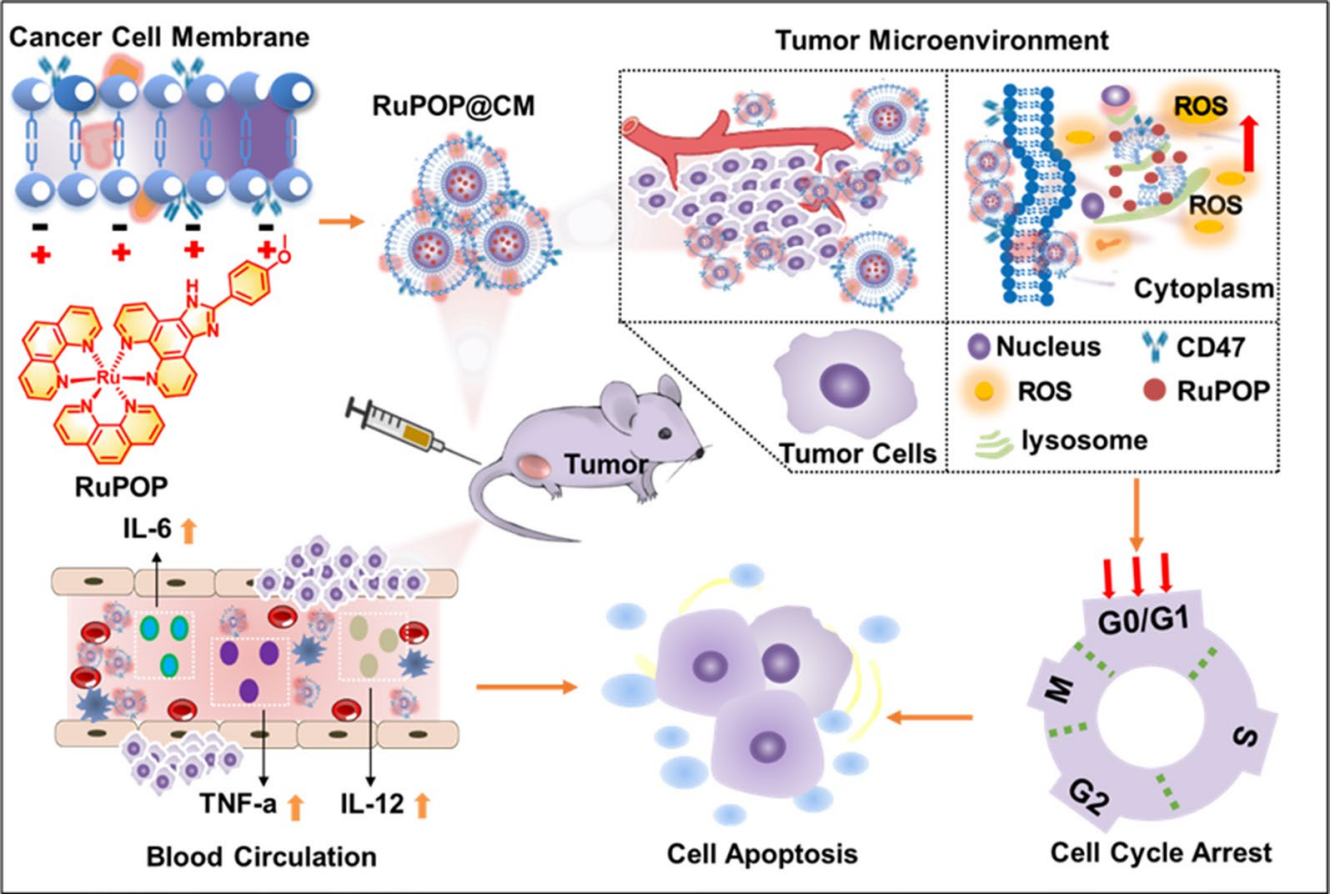
Schematic illustration of engineering cancer cell membrane-camouflaged metal complex for efficient targeting therapy of breast cancer

Herein, inspired by the various advantages of cell membrane biomimetic nanotechnology, we utilized cancer cell membranes (CM) to camouflage ruthenium polypyridyl complexes (RuPOP) and obtained a biomimetic nano-platform of RuPOP@CM. Further study results demonstrate that after camouflage decoration of cell membranes, RuPOP can reduce hemolysis, improve blood compatibility and biosafety, prolong circulation time by inhibiting phagocytosis of macrophage. Besides, RuPOP@CM can enhance the activity of cellular immune response and produce inflammatory cytokines in blood including IL-6, IL-12 and TNF-α, which is of great significance in treatment for circulating tumor cells from tumor metastasis or hematologic tumors. Breast cancer cells MDA-MB-231 and leukemia K562 have more tumor cells circulating in blood, so these two kinds of cells were selected as our targets. MDA-MB-231 cell membranes loaded with RuPOP (RuPOP@MCM) and K562 cell membranes modified with RuPOP (RuPOP@KCM) were successfully synthesized respectively. Surface camouflage modification of CM retains tumor adhesion molecules like adhesion molecules, surface antigens in nanodrugs, which makes RuPOP avoid phagocytosis by macrophage in the blood and possess it strong tumor-reaching ability. On the other hand, RuPOP@MCM can produce intracellular ROS overproduction, thereby accelerating the apoptosis and cell arrest of tumor cells to play an excellent anti-tumor effect (Scheme 1). In a word, this study provides a smart design of bioinspired nanomaterials with cancer cell membrane-camouflaged nanotechnology for metal complexes to overcome their shortcomings and enhance the cancer treatment.

## Results and discussion

### Synthesis and characterization of RuPOP@CM

**Fig. 1:**
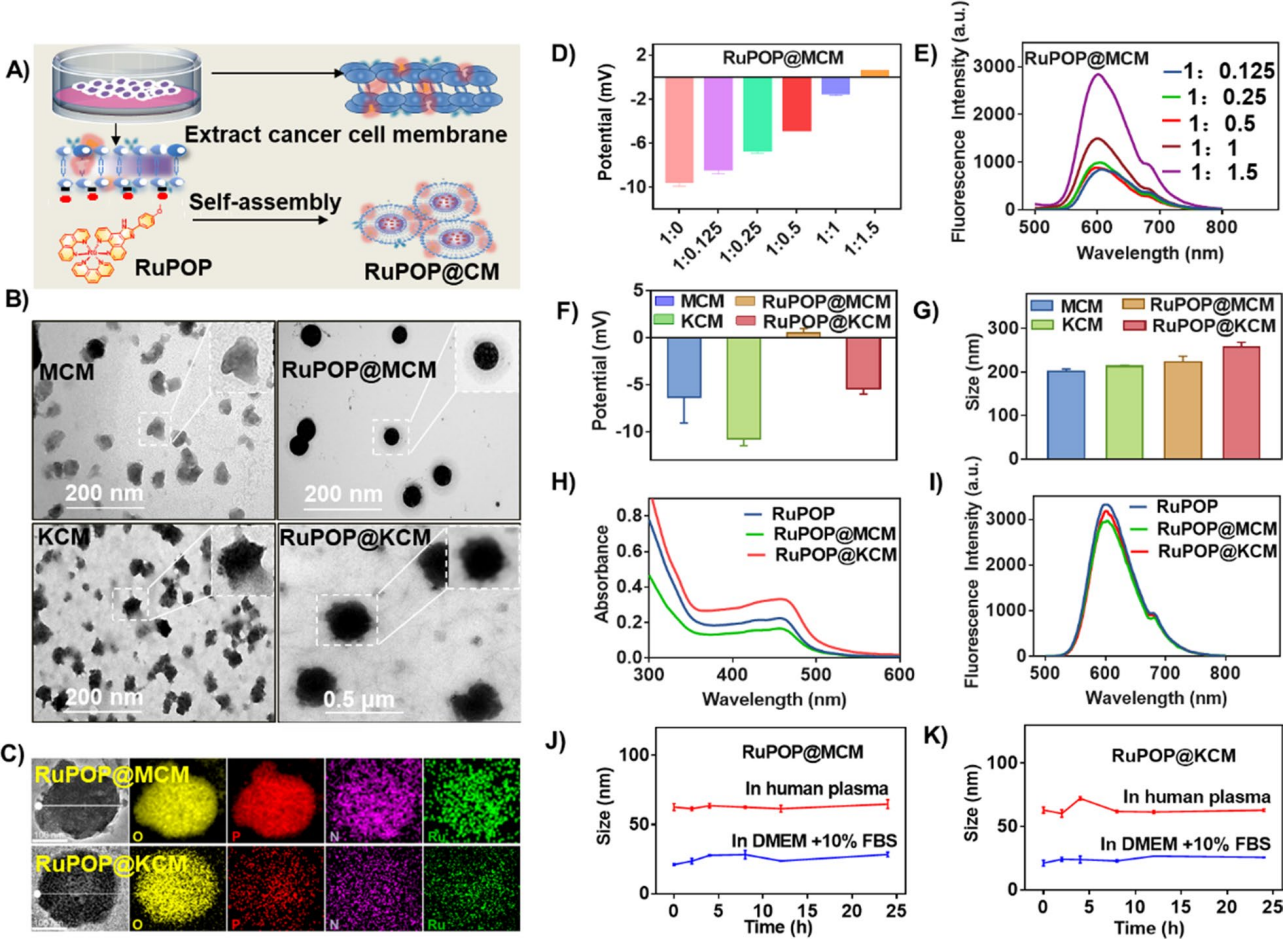
Rational design and synthesis of RuPOP@MCM. **A** Schematic illustration of the preparation of RuPOP@MCM and RuPOP@KCM. **B** TEM images of MCM, RuPOP@MCM, KCM, and RuPOP@KCM. **C** Elemental mapping of RuPOP@MCM and RuPOP@KCM. **D** Zeta potential and (**E**) fluorescence spectra of RuPOP@MCM at different mixing ratio proportions with MCM and RuPOP. **F** Zeta potential and (**G**) average hydrodynamic diameters of MCM, RuPOP@MCM (at ratio of 1:1.5), KCM, and RuPOP@KCM (at ratio of 1:1.5). **H** UV–vis spectra and (**I**) Fluorescence spectra of RuPOP, RuPOP@MCM (at ratio of 1:1.5) and RuPOP@KCM (at ratio of 1:1.5). Stability of (**J**) RuPOP@MCM and (**K**) RuPOP@KCM in DMEM with 10% FBS or human serum within 24 h

The preparation of cancer cell membrane-camouflaged RuPOP complex (RuPOP@CM) is illustrated in Fig. 1A, RuPOP@CM was prepared by repeated extrusion of the RuPOP and freshly extracted MDA-MB-231 and K562 cell membranes through polycarbonate porous membranes. In this study, RuPOP@MCM and RuPOP@KCM were successfully synthesized and characterized by microscopic and spectroscopic analysis. In Fig. 1B, TEM images demonstrated that the extracted MDA-MB-M231 and K562 cell membranes were irregular sheets with size at about 100 nm. RuPOP@MCM and RuPOP@KCM were spherical with similar size, highly monodisperse, and uniform shape comparing to the pure cell membranes. Next, the presence of O, P, and Ru signals in the final RuPOP@CM nanoparticles further confirmed the successful camouflage of cell membranes (Fig. 1C). Encouragingly, we employed an interesting exploration of the optimal ratio of RuPOP binding in CM. As expected, the potential of RuPOP@MCM reached its maximum at the volume ratio of RuPOP wrapped by cell membrane at 1:1.5 (Fig. 1D), and the fluorescence spectra also showed the same results (Fig. 1E), we further chose this ratio for subsequent experiments. In contrast, from the zeta potential, the camouflage by negative-charged CM of MDA-MB-231 and K562 cells led to increase of RuPOP@MCM and RuPOP@KCM zeta potential to 0.48 mV and − 5.3 mV respectively, compared to − 6.3 mV, − 10.8 mV of the free MCM and KCM, which may attribute to the reduced surface charge of cancer cells membranes (Fig. 1F). Additionally, hydrodynamic diameters of MCM, KCM, RuPOP@MCM, and RuPOP@KCM were 200 nm, 213 nm, 223 nm, and 257 nm, respectively (Fig. 1G). The obvious absorbance peaks of RuPOP and RuPOP@CM at 479 nm (Fig. 1H) and the fluorescence emission peaks at 600 nm (Fig. 1I) are highly coincident, indicating that RuPOP has been camouflaged by CM. Furthermore, to evaluate their dispersion and stability, we observed the size changes of MCM, KCM, RuPOP@CM, and RuPOP@KCM in different physiological environments, such as DMEM containing 10% fetal bovine serum and human serum. As shown in Fig. 1J,K, the size of these nanoparticles in both solutions was stable during the observation period of 24 h, indicating that RuPOP@CM has good stability in blood. To sum up, these results confirm that RuPOP was successfully encapsulated by cancer cell membranes.

### In vitro **anticancer efficacy of RuPOP@CM**

**Fig. 2:**
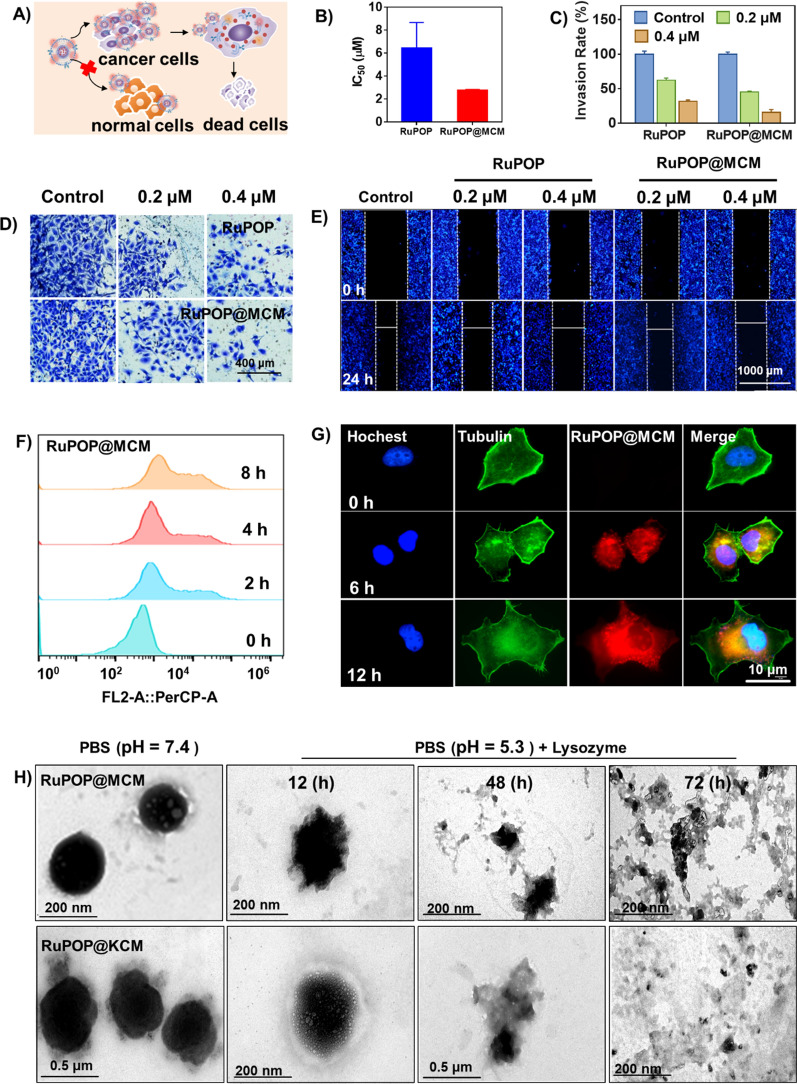
Excellent anti-tumor ability of RuPOP@MCM in vitro. **A** Scheme of RuPOP@MCM treatment to kill MDA-MB-231 tumor cells. **B** IC_50_ of MDA-MB-231cells treated with different concentrations of RuPOP and RuPOP@MCM for 72 h. **C**-**D** Anti-invasion effect assay of RuPOP or MCM@RuPOP on MDA-MB-231 cells. **E** Wound healing assay of RuPOP or RuPOP@MCM on MDA-MB-231 cells (scale bar = 100 μm). **F** Cellular uptake of RuPOP@MCM on MDA-MB-231 cells at different time. **G** Intracellular localization of RuPOP@MCM in MDA-MB-231 cells. The cytoskeleton was labeled with Alexa Fluor 488 phalloidin (green fluorescence), and nucleus was labeled with Hoechst 33,342 (blue fluorescence). **H** Morphology of RuPOP@MCM and RuPOP@KCM after incubation with lysozyme for 12 h, 48 h, 72 h

To assess the anticancer effect and safety of RuPOP@CM in vitro, we evaluated the toxicity of RuPOP, RuPOP@MCM, and RuPOP@KCM against tumor and normal cells, including MDA-MB-231 cells, K562 cells, HK-2 cells, WI-38 cells and Ect1/E6E7 cells (Fig. 2A). Comparing to RuPOP alone, RuPOP@MCM exhibited lower cytotoxicity to normal cells, such as HK-2 cells, WI-38 cells and Ect1/E6E7 cells, while it showed stronger lethality to MDA-MB-231 cells (Fig. 2B, Additional file 1: Figure S1). These results show that the camouflage decoration of cancer cell membranes can reduce the toxicity of RuPOP to normal cells and enhance its safety. Cell invasion and migration are necessary hallmarks for tumor development and metastasis. To further evaluate the anticancer activity of RuPOP@CM nano-drugs, we carried out transwell invasion and scratching experiments. As shown in Fig. 2C, D, RuPOP@MCM significantly inhibited the invasion of tumor cells at a low-toxic concentration. At the same time, RuPOP@CM can significantly inhibit the proliferation of tumor cells and form a scratch gap (Fig. 2E). The results clearly show that RuPOP@CM still has an efficient anticancer activity and excellent inhibition to tumor invasion and migration in vitro.

Moreover, cellular uptake has been regarded as a key factor in antitumor activity of medicine. Hence, we firstly detected cellular uptake of RuPOP, RuPOP@MCM and RuPOP@KCM by flow cytometry. In Fig. 2F, the cellular uptake of RuPOP@CM increased as time increased, and the uptake of RuPOP@MCM and RuPOP@KCM was higher than RuPOP alone (Additional file 1: Figure S2A-B), further indicating that the camouflage of cell membranes can increase the cellular uptake of RuPOP. By staining the cytoskeleton of MDA-MB-231 cells with Alexa Fluor 488 phalloidin, we certified that RuPOP@MCM accumulated in the cytoplasm of cells by 12 h (Fig. 2G). Besides, we also explored the intracellular translocation of RuPOP@MCM by staining nucleus and lysosomes with Hoechst 33,342 (blue) and Lyso-Tracker (green). As expected, the red fluorescence of RuPOP@MCM increased in a time-dependent method and merged well with green fluorescent signals (Additional file 1: Figure S2C), conforming that the entry of RuPOP@MCM into cells was mediated by lysosomal endocytosis. Subsequently, we simulated the systemic circulation environment of human body (PBS at pH 7.4) and the acidic environment in cell lysozyme (PBS at pH 5.3 with lysozyme (1 mg mL^− 1^), and observed the RuPOP release from RuPOP@MCM and RuPOP@KCM under different conditions. As shown in Figure S3, as time increased, RuPOP@MCM gradually released in the systemic circulation environment of human body (PBS at pH 7.4). In contrast, RuPOP@MCM in the acidic environment in cell lysozyme (PBS at pH 5.3 with lysozyme) had released a lot at 12 h incubation much more than in PBS at pH 7.4, which indicates that lysosomes are important organelles for the intracellular localization of RuPOP@MCM, and RuPOP@MCM was better released in lysozyme environment. RuPOP@MCM and RuPOP@KCM were homogeneous and highly dispersed in PBS at pH 7.4 (Fig. 2H). Under incubation of lysozyme solution, these two nanoparticles showed shrinkage and cavitation after 12 h. As time increased, these nanoparticles gradually lost their complete spherical appearance and continued expansion and rupture in the lysosomal environment, then completely broken at 72 h. This phenomenon suggests that the biological interaction between biological enzyme in cell lysozyme and cell membranes, thus leading to the disintegration of RuPOP@CM and RuPOP release. To sum up, these results demonstrate that lysosomes are important organelles for the intracellular localization of RuPOP@MCM, directedly affecting the uptake of nano-drugs and the release of RuPOP.

### Hemocompatibility, pharmacokinetic property and immunogenicity in vivo of RuPOP@CM

**Fig. 3:**
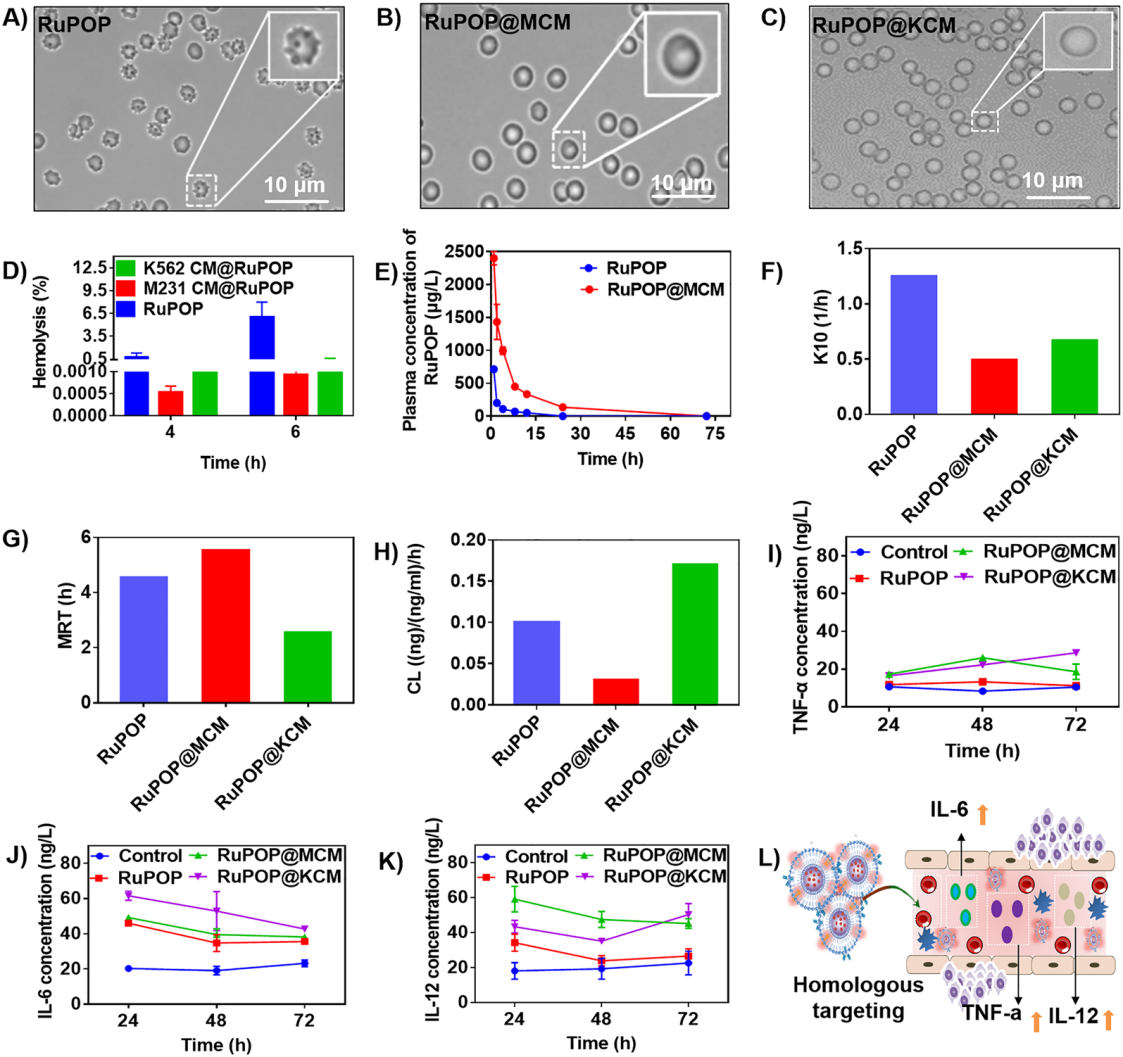
Hemocompatibility and immune activity of RuPOP@CM in vitro. Microscopy image of red blood cells treated with (**A**) RuPOP, (**B**) RuPOP@MCM and (**C**) RuPOP@KCM for 6 h. **D** Hemolysis detection of RuPOP, RuPOP@MCM and RuPOP@KCM. **E** The content of RuPOP in plasma versus time after intravenous injection of RuPOP and RuPOP@MCM. Pharmacokinetic index (**F)** K10, (**G)** MRT (**H**) CL of RuPOP, RuPOP@MCM, and RuPOP@KCM. Cytokine levels of (**I**) TNF-α, (**J**) IL-6, and (**K**) IL-12 in serum from mice after being treated with RuPOP, RuPOP@MCM and RuPOP@KCM for 24 h, 48 h, and 72 h respectively (*n* = 5 mice per group). **L** Scheme illustration of activated immune activity induced by RuPOP@MCM

Hemocompatibility of metal complex is an important factor that should be considered in evaluation of biosafety in medicine. We evaluated the biosafety of RuPOP@CM in mice. From photos of red blood cells Fig. 3A-C, we can see directly that the surface of red blood cells treated with RuPOP appeared small pores or even damaged, while there were no significantly morphological changes with incubation by RuPOP@CM. Furthermore, the hemolysis rate of RuPOP was higher than RuPOP@MCM and RuPOP@KCM nanoparticles (Fig. 3D). The hemolysis rate of RuPOP@MCM and RuPOP@KCM nanoparticles were all kept under 5% at different times. These results indicate that the hemocompatibility of RuPOP was significantly increased due to the camouflage modification of cell membranes, which promotes the application of the biomimetic medicine in clinical.

 More importantly, quick clearance in blood circulation is also another serious shortcoming of medicine that can’t be ignored. Therefore, to verify whether bionic camouflage of cell membrane can ameliorate these defects, we performed pharmacokinetic analysis of RuPOP and RuPOP@MCM. As shown in Fig. 3E, the blood content of RuPOP@MCM nanoparticle was higher than free RuPOP, we also found that RuPOP alone was cleared quickly as time increased. According to the pharmacokinetic parameters calculated by the fitting equation, after bionic camouflage modification of cell membranes, the elimination rate (K10) of RuPOP@MCM in blood decreased (Fig. 3F). In addition, RuPOP@MCM also significantly enhanced the elimination half-life (elimination period, T1 shock 2 β), maximum plasma concentration (Cmax), area under the curve (AUC 0–80 h), average retention time (MRT) and decreased clearance (CL) value (Fig. 3G,H) comparing with free RuPOP. For example, the blood clearance rate of RuPOP@MCM nanoparticles (CL = 0.03 ng/(ng/mL)/h) was less than RuPOP (CL = 0.10 μg/(ng/mL)/h). Interestingly, half-life of RuPOP@MCM nanoparticles (t_1/2_=2.4 h) was higher than RuPOP (t_1/2_=1.6 h), this phenomenon indicates that the blood circulation time of RuPOP@CM was prolonged (Additional file 1: Table S1, S2 and S3). All of these indexes are consistent with two compartment pharmacokinetics. Therefore, these results further demonstrate that bionic camouflage decoration of cell membranes can enhance the blood circulation time and improve pharmacokinetics of RuPOP to a certain extent.

In addition, we suggest that it may probably because of a large number of surface antigens and tumor adhesion molecules retained on the cancer cell membranes that trigger a stronger inflammatory response of macrophages. We further monitored the changes of cytokines TNF-α (important marker of cellular immune activation), interleukin-6 (IL-6, important marker of humoral immune activation) and interleukin-12 (IL-12, important marker of innate immune activation) in serum of mice after injection with RuPOP@CM nanoparticles. As shown in Fig. 3I, J, K, the release of three cytokines induced by RuPOP@MCM and RuPOP@KCM nanoparticle were higher than free RuPOP group. In addition, the release of IL-6 induced by RuPOP@MCM and RuPOP@KCM nanoparticles was lower than LPS group at 72 h (Additional file 1: Figure S4). Thus, we suggest that it may probably because of a large number of surface antigens and tumor adhesion molecules retained on the cancer cell membranes that trigger a stronger inflammatory response of macrophages, which has an excellent advantage in the treatment of tumor, especially against to the circulating tumor cells from tumor metastasis or hematologic tumors (Fig. 3L).

**Fig. 4:**
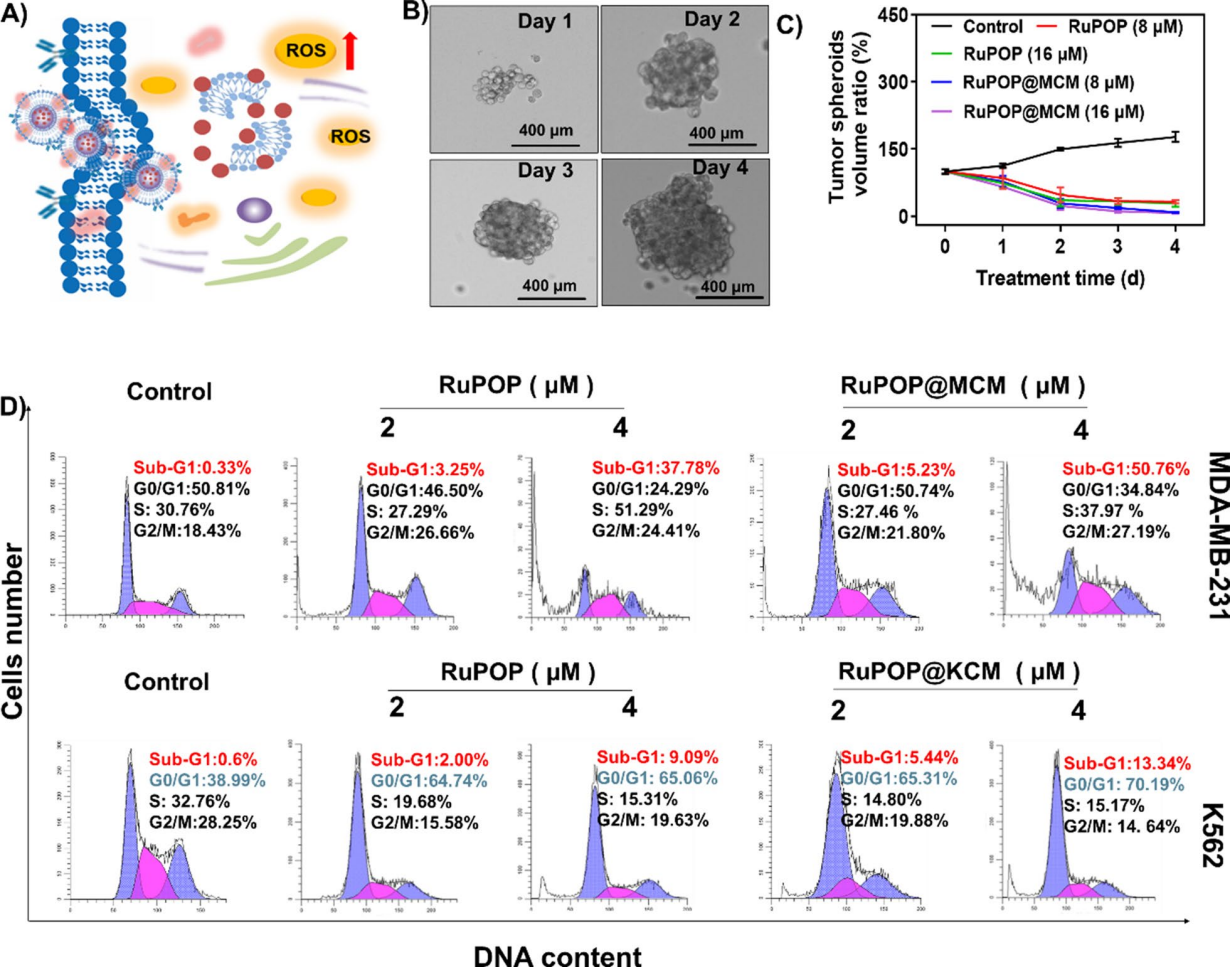
The anticancer mechanism of RuPOP@CM in vitro. **A** Schematic illustration of RuPOP@CM triggering excess ROS generation and accelerating cell apoptosis and cell cycle arrest. **B** The formation of MDA-MB-231 tumor spheroids. **C** Inhibition curves of different concentrations of RuPOP and RuPOP@MCM on tumor spheroids. **D** Flow cytometric analysis on the cell cycle of MDA-MB-231 cells and K562 cells after treatment with different concentrations of RuPOP@MCM and RuPOP for 72 h

### The anticancer mechanism of RuPOP@CM nanoparticles

Based on the excellent antitumor effect of RuPOP@CM in vitro, we then carried out the study to briefly explain their potential antitumor mechanism (Fig. 4A). Firstly, more literatures have reported that when the tumor spheroids grew to more than 200 μm in diameter, it had a microenvironment similar to that of tumor tissue in vivo. In spired by this, we successfully established a tumor sphere model of MDA-MB-231 cells in vitro, and made it grow into a diameter of about 200 μm on the fourth day to simulate tumor tissue (Fig. 4B). Then we incubated the tumor spheroids with different concentrations of RuPOP@MCM and RuPOP, and measured the volume of tumor spheroids continuously. The experimental results showed that the tumor spheroids in control group increased rapidly as time increased, while different concentrations of RuPOP@MCM nanoparticles could well suppress the growth of tumor spheroids, especially in RuPOP@MCM, further indicates that the inhibitory effect of RuPOP@MCM was higher than RuPOP alone (Fig. 4C). We suggest that the different inhibitory effect of RuPOP@MCM and RuPOP on tumor spheroids may attribute to the absorption of cell membranes by tumor spheroids. In conclusion, the bionic modification of cell membrane improves the permeability of RuPOP in tumor spheres.

**Fig. 5:**
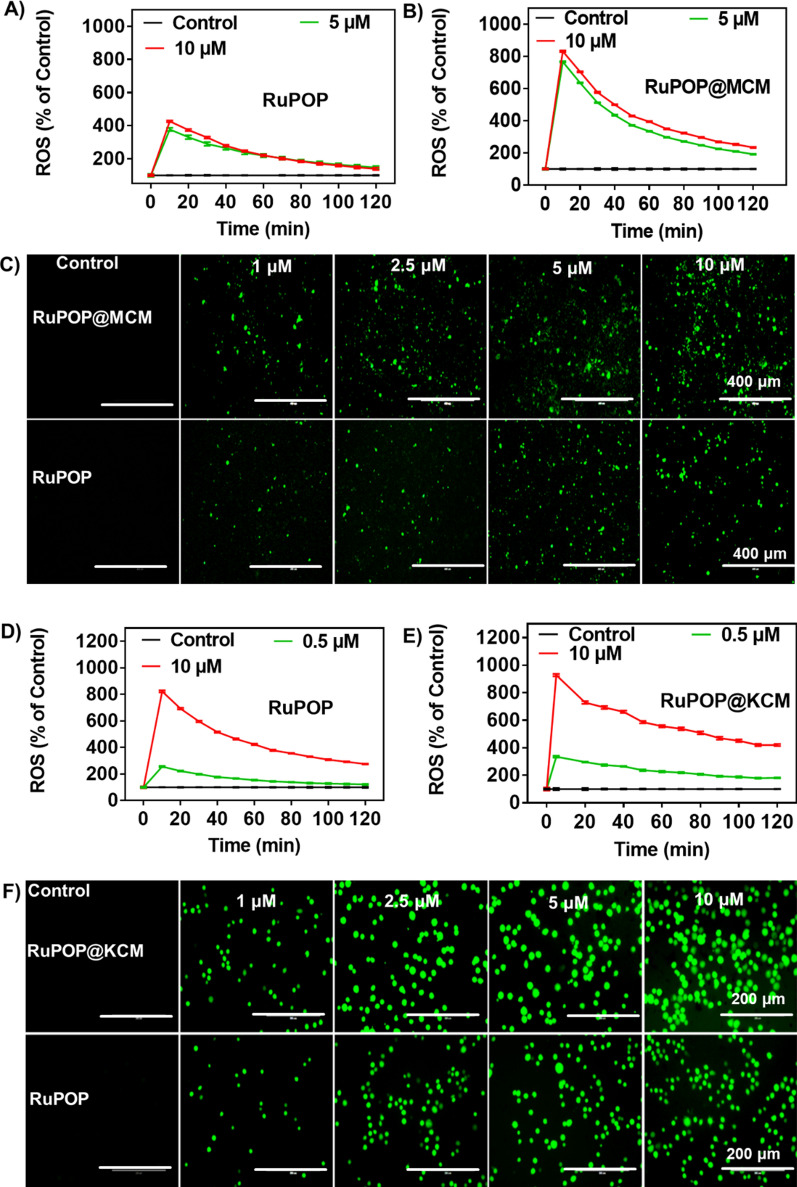
The anticancer mechanism of RuPOP@CM in vitro. **A**,**B**,**C** ROS generated by different concentrations of RuPOP and RuPOP@MCM on MDA-MB-231 cells analyzed by DCF-DA assay. **D**,**E**,**F** ROS generated by different concentrations of RuPOP and RuPOP@KCM on K562 cells analyzed by DCF-DA assay

Cell apoptosis and cycle arrest are vital ways to inhibit tumor proliferation in vitro. Flow cytometry showed that RuPOP@MCM and RuPOP@KCM could effectively induce apoptosis of MDA-MB-231 and K562 cells (Fig. 4D). For example, RuPOP@MCM (0–4 µM) increased the apoptosis of MDA-MB-231 cells, which confirmed that the proportion of apoptotic cells increased from 0.33 to 50.76%. RuPOP@KCM (0–4 µM) caused an enhancement in the proportion of apoptotic cells from 0.6 to 13.34%, and simultaneously increased G0/G1 cell population from 38.99 to 70.19%. These results indicate that RuPOP@MCM and RuPOP@KCM inhibited the development of tumor cells by inducing G0/G1 phase arrest. Studies have shown that chemotherapeutic drugs disrupted the stable level of intracellular ROS, resulting in damage to the function of biological macromolecules, then induced cell apoptosis. Then, fluorescent probe DCFH-DA was used to detect the changes of ROS levels in MDA-MB-231 cells (Fig. 5A, B, C) treated with RuPOP@MCM and RuPOP, K562 cells (Fig. 5D, E, F) treated with RuPOP@KCM and RuPOP. The intracellular fluorescence intensity enhanced with the increasing concentration of RuPOP, RuPOP@MCM and RuPOP@KCM nanoparticles. Besides, we also detected the cell apoptosis and the mitochondrial membrane potential (MMP, Δψm) induced by RuPOP@MCM (Additional file 1: Figure S5), we found that RuPOP@MCM significantly increased the early apoptosis and late apoptosis cancer cells. The MMP is decreasing in a dose-dependent manner, which reflects increase of the green fluorescence ratio. In summary, these results suggest that RuPOP@MCM and RuPOP@KCM exhibited significant antitumor activity by enhancing intracellular ROS levels, inducing DNA damage to accelerate cell apoptosis and cell cycle arrest.

### In vivo **anticancer efficacy of RuPOP@MCM**

**Fig. 6:**
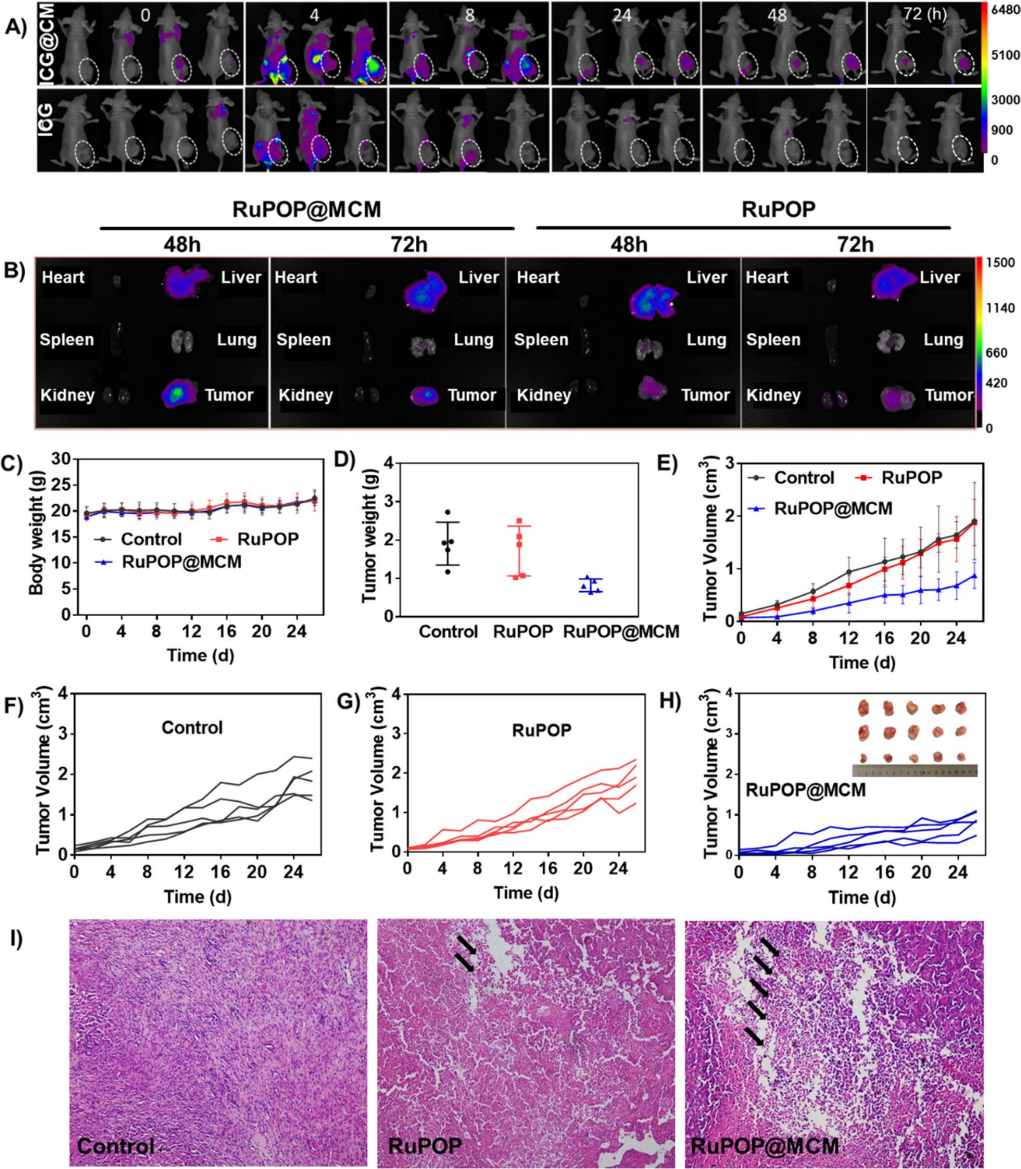
Cell membrane camouflage decoration of RuPOP for efficient targeting therapy of breast cancer in vivo. Fluorescence imaging monitors the accumulation and distribution of RuPOP@MCM or RuPOP in (**A**) MDA-MB-231 cells xenografts nude mice and (**B**) main organs at different time points. **C** Body weight, and (**D**) tumor weight of nude mice after treatment with RuPOP@MCM and RuPOP. **E**,**F**,**G**,**H** Changes in tumor volume of the control, RuPOP@MCM and RuPOP group within 3 weeks. **I** H&E staining of tumor sections after treatment with RuPOP@MCM, RuPOP for three weeks, tumor necrosis was indicated by the black arrows

Encouraged by the satisfactory hemocompatibility and biosafety, we then subsequently investigated the tumor penetration and tissue distribution of RuPOP@MCM in MDA-MB-231 tumor-bearing nude mice by live imager. To enhance the efficiency of tissue imaging, we chose indolyanine green (ICG) as the Near infrared fluorescence indicator labeled the RuPOP@MCM nanoparticles. As shown in Fig. 6A, after injection with RuPOP and RuPOP@MCM for 4 h, the fluorescence intensity of RuPOP@MCM group in tumor region was significantly higher than RuPOP group. Especially, after three days, the fluorescence intensity of RuPOP@MCM in tumor region of mice was still stronger than RuPOP group, which alleviates the problem of insufficient distribution of RuPOP in the tumor region. Besides, in order to accurately determine the distribution of drugs in various tissues, the fluorescence intensity of each organ tissues of mice was detected at 48 h, 72 h respectively (Fig. 6B), which further confirms the higher accumulation of RuPOP@MCM in tumor tissues than RuPOP alone. The above results suggest that the camouflage modification of cell membranes can not only improve the targeted ability of RuPOP to tumor tissue in vivo, but also enhance its accumulation in tumor region, thus performing strong antitumor effect in vivo.

Next, we further assessed the antitumor ability of RuPOP@MCM in MDA-MB-231 tumor-bearing nude mice. In Fig. 6C, comparing to control group, there existed no significant difference in body weight of mice between group RuPOP and RuPOP@MCM group, demonstrating that RuPOP@MCM had no obvious toxic side effect to mice. What’s more, from the tumor weight and volume (Fig. 6D-H), we found that RuPOP@MCM inhibited the growth of the MDA-MB-231 tumors, as verified by the decrease in tumor volume and tumor growth ratio in RuPOP@MCM treated group, further confirming the excellent and effective antitumor ability of RuPOP@MCM in vivo. Treatment with RuPOP exhibited lower tumor inhibition than RuPOP@MCM, which verifies the importance of the cell membranes mediated tumor homing ability for effective cancer treatment. Additionally, H&E staining was further performed to confirm the anticancer ability. The cell density of tumor sections in the control group was very close, indicating that MDA-MB-231-bearing nude mice were undergoing malignant tumor progression. Mice treated with RuPOP@MCM showed evident nuclear condensation and decreased cell density in the tumor sites (Fig. 6I), further illustrating the excellent antitumor ability of RuPOP@MCM in vivo. We also performed histological analysis in main organs to further evaluate the biosafety of RuPOP@MCM. The slices of major organs didn’t detect obvious inflammation or other changes induced by RuPOP@MCM, which further proves its higher safety and lower toxicity (Additional file 1: Figure S6). Thus, these results demonstrate that the biomimetic camouflage modification of cell membranes can improve the targeted and permeated ability of RuPOP to tumor tissue in vivo, thus enhancing the antitumor effects of RuPOP.

## Conclusion

Rational modification of metal complexes that could enhance the biocompatibility and decrease clearance of compounds in blood circulation to accurately recognize and eradicate tumor cells, is of great significance and application potential for metal complexes in cancer treatment. Cancer cell membranes retain a large number of surface antigens and tumor adhesion molecules on the surface, which can be used to modify the metal complex and overcome its defects. In this study, we used cell membrane biomimetic nanotechnology to camouflage RuPOP metal complex to obtain the engineering cell membrane-camouflaged metal complex RuPOP@CM. On the one hand, the hemocompatibility and biosafety of RuPOP increased significantly due to cell membranes camouflage, and the engineering camouflage modification of cell membranes can effectively enhance the blood circulation time of RuPOP to prevent phagocytosis of macrophages. Interestingly, because of a large number of surface antigens and tumor adhesion molecules retained on the cancer cell membranes, which triggered a stronger inflammatory response of macrophages, further indicating that RuPOP@CM may induce an immune response in vivo. On the other hand, RuPOP@MCM increased intracellular ROS levels, broke the redox balance in tumor cells, thus accelerating the apoptosis and cycle arrest of cells to perform excellent antitumor efficiency. More importantly, the outstanding antitumor ability in vivo of RuPOP@MCM was verified, engineering camouflage modification of cell membranes endows RuPOP with compatibility to target tumor tissue and increases its accumulation in tumor sites, thus enhancing the antitumor effects of RuPOP. Therefore, this work provides a smart design of bioinspired engineering nanoplatform with cell membranes camouflaging nanotechnology for metal complex to overcome their shortcomings and enhance the cancer treatment.

## Experimental section

### Material and methods

[Ru(phen)_2_-p-MOPIP] (PF_6_)_2_·2H_2_O (RuPOP) was synthesized as previously described in previous report [5]. Thiazolyl blue tetrazolium bromide (MTT), ICG, DMEM media, Hoechst 33,342 were purchased from Sigma-Aldrich. Lyso-tracker Green was purchased from Life Technologies. Matrix glue was purchased from Corning.

#### Cell line and cell culture

Human breast cancer cell line of MDA-MB-231, human myelogenous leukemia cell line of K562 and human normal breast cell line of Hs578bst were cultured in DMEM containing 10% FBS and 1% penicillin-streptomycin solution (Beyotime, Code No. C0222).

### Preparation of CM

Firstly, cancer cells (MDA-MB-231 and K562 cells) were centrifuged, the collected cell deposits were washed with PBS buffer for 3 times. After that, the washed cell deposits were suspended in hypotonic lysis buffer and grounded with a homogenizer, centrifuged again at 3000 rpm, then collected the supernatant and centrifuged at 12,000 rpm. Finally, the supernatant was collected and transferred to another test tube, which was centrifuged at 38,000 rpm to collect cell deposits. The collected cell deposits were then washed with 10 mM Tris-HCl and 1 mM EDTA, and centrifuged at 38,000 rpm. The final cell deposits were suspended in PBS, which would be extruded serially through 400 nm and 200 nm polycarbonate porous membranes respectively by using an Avanti mini extruder (Avanti polar grease).

### Preparation of RuPOP@CM

RuPOP was dissolved in DMSO with a concentration of 5 mg/mL. The cell membrane (28.8 µg in 400 µL PBS) and the prepared RuPOP (3 mg in 600 µL DMSO) at a volume ratio of 1:1.5 were treated with ultrasound at 37 kHz for 2 h, and then extruded to prepare RuPOP@CM. The mechanical force produced by extrusion promoted the fusion of cell membrane and RuPOP, then RuPOP wrapped in cell membranes. Further, the final product of RuPOP@CM was used in subsequent experiments.

### Characterization of RuPOP@CM

The size and zeta potential of RuPOP@MCM and RuPOP@KCM were characterized by Nano-ZS Instruments (Malvern Instruments Co., Ltd., UK), and its morphology was observed by transmission electron microscope (TEM, JEM-2100 F, JEOL, Japan). Additionally, the UV-vis-NIR spectrum was detected by the UV-Vis floor near-infrared spectrophotometer at range of 300 ~ 600 nm, and its fluorescence spectrum was also detected by a fluorescence spectrophotometer with a wavelength at range of 500 ~ 800 nm. The concentration of RuPOP in cell membranes was determined by ICP-MS.

### Stability of RuPOP@CM

Approximately 0.5 mL of PBS, 0.5 mL of DMEM supplemented with 10% FBS and 0.5 mL human serum were mixed with equal volume of RuPOP@MCM or RuPOP@KCM respectively. During different incubation periods, the sizes of RuPOP@MCM and RuPOP@KCM were determined by Zetasizer Nano ZS particle analyzer.

### Hemolysis rate of RuPOP@CM

Hemolysis rate of RuPOP@CM was determined to evaluate its biocompatibility in blood. The red blood cells were treated with PBS, RuPOP, RuPOP@MCM, RuPOP@KCM for 2 h, respectively. The red blood cells treated with PBS and Triton X-100 were used as negative and positive control, respectively.[35] Then, the red blood cells were rotated downward and the absorbance of the supernatant was measured at 540 nm. The hemolysis rate was calculated according to the following formula.

Hemolysis rate (%) = (A_Sample_-A_Negtive Control_) / (A_Positive Control_ -A_Negative Control_) *100%. In order to study the cell morphology of the collected red blood cells, we place each sample on a piece of glass, and observe it with a phase-contrast microscope (Life Technologies, EVOS FL AUTO).

### Pharmacokinetic study of RuPOP@CM

Fifteen female SD mice (100 g per mouse) were separated into three groups, which treated with RuPOP, RuPOP@MCM and RuPOP@KCM, via intravenous injection with an equivalent dose of 1 mg/kg RuPOP (200 µL per mouse). Then blood samples were collected at different time points (0 h, 1 h, 2 h, 4 h, 8 h, 12 h, 24 h, 48 h, 72 h). The serum of blood samples was nitrified and the Ru contents were determined by ICP-MS. The data fitting and calculations of related pharmacokinetic parameters were realized by Winonlin 3.3 software.

### **Immunogenicity of RuPOP@CM** in vivo

Forty-eight female Balb/c mice (18–22 g per mouse) were randomly separated into 4 groups and treated with saline, RuPOP, RuPOP@MCM, RuPOP@KCM respectively, via intravenous injection with an equivalent dose of 1 mg/kg RuPOP (injection volume: 200 µL). Mice in control group were injected with saline at a dose of 200 µL per mouse. Then, blood samples were collected at 24 h, 48 h, and 72 h. Then, the concentrations of TNF-α, IL-6, and IL-12 in serum were examined by ELISA kits.

### In vitro**anticancer efficacy of RuPOP@CM**

MDA-MB-231 cells, K562 cells, HK-2 cells, Ect1/E6E7 cells and WI-38 cells were plated on 96-well plate (2000 cells per well), and incubated with different concentration of RuPOP@CM (1.25 µM, 2.5 µM, 5 µM, 10 µM, 20 µM, 40 µM) for 72 h, then assessed the cytotoxicity by MTT assay [36–38] and calculated the IC_50_ value of RuPOP@CM.

### Examination of cell migration, invasion

The MDA-MB-231 cells were inoculated in 6-well plate (5 × 10^4^ cells/mL), then removed the medium and starved cells (medium containing 5% FBS) for 6 h. When cells cover the bottom of the plate, then make the scratch with sterile spear (200 µL) and wash the cells with PBS three times. Cells were incubated with RuPOP and RuPOP@MCM nanoparticles of different concentrations (0.2 µM and 0.4 µM) for 24 h. Changes in the gap were recorded with a microscope, and the degree of closure was indicated by the width of the gap.

### Flow cytometric analysis

Cells were cultured in a 6 cm dish (20 × 10^4^ cells/mL) for 24 h, then treated with different concentrations (2 µM, 4 µM, 8 µM) of RuPOP@MCM or RuPOP@KCM, the cells were washed with PBS. Finally, cells were fixed with at -20 ℃ overnight. [39]The fixed cells were washed and stained with propidium 500 µL iodide (PI) for 1 h at 4 ℃. The stained cells were determined by flow cytometer (Epics-XL, Beckman Coulter) to explore cell cycle distribution, followed by data analysis using MultiCycle software.

### Measurement of intracellular ROS generation

ROS generated in MDA-MB-231 cells and K562 cells with RuPOP@MCM and RuPOP@KCM treatments was determined by a fluorescent probe of DCFH-DA. Firstly, MDA-MB-231 cells were inoculated in 96-well plate (20 × 10^4^ cells/mL, 100 µL) [40, 41]. Next day, the supernatant was discarded, and cells were incubated with 100 µL PBS containing DCFH-DA probe (10 µM) for 0.5 h. Then, different concentration of RuPOP, RuPOP@MCM and RuPOP@KCM were added, the absorbance value of cells in each treatment group was detected immediately under a fluorimeter (Ex = 488 nm, Em = 525 nm), and monitored continuously for 2 h.

### Cellular uptake and trafficking of RuPOP@MCM

The absorption of RuPOP and RuPOP@MCM nanoparticles in MDA-MB-231 cells was measured according to the fluorescence intensity of RuPOP. Cells were inoculated in 6-well plate (10 × 10^4^ cells/mL). After the incubation, RuPOP and RuPOP@MCM nanoparticles were added to the 6-well plate and incubated for 2 h, 4 h, 6 h and 8 h with cells, respectively [42]. After that, the supernatant was removed, and cleaned by PBS solution. Then, cells were digested with trypsin and collected in the centrifuge tube, and analyzed the fluorescence intensity of RuPOP in cells to analyze the cellular uptake of RuPOP@MCM.

To detect the intracellular translocation of RuPOP@MCM, the MDA-MB-231 cells (8 × 10^4^ cells/mL) were inoculated in a 2 cm dish. Next day, cells were labeled lysosome with Lyso Tracker green fluorescent probe or stained cytoskeleton with Fluor 488 phalloidin (green) for 2 h, and labeled the nuclear with Hochest 33,342 dye (blue) for 1 h. Then, RuPOP@MCM were added and incubated for 0 h, 2 h, 4 h, 6 h and 8 h respectively. Cells were cleaned to remove residual drugs in the medium. The fluorescence signal of drugs in cells was monitored in real time under fluorescence microscope. The nanomaterials emit red fluorescence in cells due to the loading of RuPOP, and the localization of nanomaterials in cells was analyzed by monitoring the overlap of drug red fluorescence with lysosomal, cytoskeleton and nuclear. At the same time, the absorption efficiency of nanodrugs in cells was evaluated by the intensity of red fluorescence in cells at different time points.

### Morphology changes of RuPOP@CM in lysozyme

RuPOP@MCM and RuPOP@KCM (with concentration of 4 µM) were mixed with PBS solution at pH 7.4 or PBS solution at pH 5.3 with lysozyme (1 mg/mL) respectively, and incubated in a constant temperature at 37 ℃ for 12 h, 48 h and 72 h. At the end of the experiment time point, the incubated nanoparticles were analyzed by TEM to evaluate the microscopic morphology changes of RuPOP@MCM and RuPOP@KCM.

### Inhibitory effect of RuPOP@MCM against MDA-MB-231 multicellular tumor spheroids

MDA-MB-231 multicellular tumor spheroids were cultured in 6-well plates and treated with different concentrations of RuPOP or RuPOP@MCM (with concentration of 8 µM, 16 µM) for 4 days [43, 44]. The length and width of MDA-MB-231 multicellular tumor spheroids were measured and recorded every day by microscopy to evaluate the inhibitory effect of RuPOP@MCM.

### In vivo antitumor activity of RuPOP@MCM

For the establishment of the MDA-MB-231 xenograft Balb/c-nude mice model, MDA-MB-231 cells (1 × 10^6^ cells per mouse) suspended in DMEM were subcutaneously injected into the armpit of the mice. When the tumor volume reached 70 mm^3^, MDA-MB-231 xenograft mice were randomly divided into three groups (n = 5 per group) and intravenously injected with saline, RuPOP (1 mg/kg), and RuPOP@MCM (1 mg/kg) every other day. Body weights and tumor sizes of each mouse were also measured every day within 26 days, and the mice were euthanized in the 26th day. Tumors were weighed and photographed. Tumor and major organs were collected and fixed in 4% paraformaldehyde.

### **Distribution of RuPOP@MCM** in vivo

Indolyanine green (ICG) was used as a fluorescence indicator to label the RuPOP@MCM nanoparticles [45]. Real-time imaging in vivo was performed to identify the biodistribution of RuPOP@MCM in MDA-MB-231 xenograft bearing nude mice. Dynamic fluorescence imaging was performed by collecting the NIR signal of ICG in mice via a live imaging system, with the observation time at 4 h, 8 h, 24 h, 48 h, and 72 h after injection. At the end of the 48 h and 72 h, mice were euthanized, the main organs and tumor of mice were subjected to ex vivo imaging.

#### Statistical analysis

All experiments in this study were examined in triplicate. Data were represented as mean ± standard deviation (SD). The difference between control and experimental groups was analyzed by the one-way analysis of variance (ANOVA) method. Differences indicated as P < 0.05 (*) or P < 0.01 (**) were considered statistically significant.

## Supplementary Information

** Additional file 1: Figure S1.** RuPOP@CM exhibits excellent anti-tumor ability in vitro. A) IC50 of K562 cells treated with different concentrations of RuPOP and RuPOP@KCM for 72 h. B) Cell viability of RuPOP@MCM and RuPOP treatment on MDA-MB-231 cells. C) Cell viability of RuPOP@KCM and RuPOP treatment on K562 cells K562 cells. Cell viability of RuPOP@MCM and RuPOP treatment on D) HK-2 cells, E) Ect1/E6E7 cells and F) WI-38 cells.** Figure S2.** Cellular uptake of RuPOP and KCM@RuPOP in MDA-MB-231 cells at different time. A) Cellular uptake of RuPOP and RuPOP@KCM on MDA-MB-231 cells at different times. B) Cellular uptake of RuPOP@MCM, RuPOP and RuPOP@KCM in MDA-MB-231 cells by determination the fluorescence intensity of RuPOP. C) Translocation of RuPOP@MCM in MDA-MB-231 cells.** Figure S3. **Release concentration of RuPOP after incubation with PBS at pH 7.4 and PBS at pH 5.3 with lysozyme for 12 h.** Figure S4. **IL-6 in serum from mice after being treated with RuPOP, RuPOP@MCM, RuPOP@KCM and LPS for 72 h respectively (n = 3 mice per group).** Figure S5. **Anticancer mechanism of RuPOP@CM in vitro. A) Cell apoptosis induced by RuPOP@MCM, B) Mitochondrial membrane potential in MDA-MB-231 cells treated with RuPOP@MCM.** Figure S6. **H&E staining image of main organs from different groups. Mice were treated with saline, RuPOP and RuPOP@MCM (1 mg/kg) through intravenous injection for 26 days.** Table S1. **Pharmacokinetic parameters of RuPOP. ** Table S2.** Pharmacokinetic parameters of RuPOP@MCM. ** Table S3.** Pharmacokinetic parameters of RuPOP@KCM.

## Abbreviations

RuPOP: Ruthenium polypyridyl complex
RuPOP@CM: Cancer cell membrane-camouflaged RuPOP
RuPOP@MCM: MDA-MB-231 cancer cell membrane-camouflaged RuPOP
RuPOP@KCM: K562 cancer cell membrane-camouflaged RuPOP
ROS: Reactive oxygen species
DMSO: Dimethyl sulfoxide
DMEM: Dulbecco’s modified eagle medium
FBS: Fetal bovine serum
MTT: Thiazolyl blue
ICP-MS: Inductively coupled plasma mass spectrometry
PI: Propidium iodide
H&E: Hematoxylin and Eosin
