# Supplementary material for: Engineering cancer cell membrane-camouflaged metal complex for efficient targeting therapy of breast cancer
Source: J Nanobiotechnology. 2022 Sep 5;20:401. doi: 10.1186/s12951-022-01593-5 (PMC9446690; doi:10.1186/s12951-022-01593-5)
Supplement: Supplementary file 1 — Additional file 1: Figure S1. RuPOP@CM exhibits excellent anti-tumor ability in vitro. A) IC50 of K562 cells treated with different concentrations of RuPOP and RuPOP@KCM for 72 h. B) Cell viability of RuPOP@MCM and RuPOP treatment on MDA-MB-231 cells. C) Cell viability of RuPOP@KCM and RuPOP treatment on K562 cells K562 cells. Cell viability of RuPOP@MCM and RuPOP treatment on D) HK-2 cells, E) Ect1/E6E7 cells and F) WI-38 cells. Figure S2. Cellular uptake of RuPOP and KCM@RuPOP in MDA-MB-231 cells at different time. A) Cellular uptake of RuPOP and RuPOP@KCM on MDA-MB-231 cells at different times. B) Cellular uptake of RuPOP@MCM, RuPOP and RuPOP@KCM in MDA-MB-231 cells by determination the fluorescence intensity of RuPOP. C) Translocation of RuPOP@MCM in MDA-MB-231 cells. Figure S3. Release concentration of RuPOP after incubation with PBS at pH 7.4 and PBS at pH 5.3 with lysozyme for 12 h. Figure S4. IL-6 in serum from mice after being treated with RuPOP, RuPOP@MCM, RuPOP@KCM and LPS for 72 h respectively (n = 3 mice per group). Figure S5. Anticancer mechanism of RuPOP@CM in vitro. A) Cell apoptosis induced by RuPOP@MCM, B) Mitochondrial membrane potential in MDA-MB-231 cells treated with RuPOP@MCM. Figure S6. H&E staining image of main organs from different groups. Mice were treated with saline, RuPOP and RuPOP@MCM (1 mg/kg) through intravenous injection for 26 days. Table S1. Pharmacokinetic parameters of RuPOP. Table S2. Pharmacokinetic parameters of RuPOP@MCM. Table S3. Pharmacokinetic parameters of RuPOP@KCM. [file 12951_2022_1593_MOESM1_ESM.docx]

**Additional file 1**

for

**Engineering Cancer Cell Membrane-Camouflaged Metal Complex f****or Efficient Targeting Therapy of Breast Cancer**

Xiaoying Li ^a, #^, Yanzi Yu ^a, #^, Qi Chen ^a^, Jiabao Lin ^a^, Xueqiong Zhu ^b^, Xiaoting Liu ^c^, Lizhen He ^a,^ *, Tianfeng Chen ^a^, Weiling He ^c,^ *

^a^ Department of Neurology and Stroke Center, The First Affiliated Hospital, Department of Chemistry, Jinan University, Guangzhou 510632, China.

^b^ Department of Obstetrics and Gynecology, the Second Affiliated Hospital of Wenzhou Medical University, Wenzhou, China.

^c^ Department of Gastrointestinal Surgery, The First Affiliated Hospital, Center for Precision Medicine, Sun Yat-sen University, Guangzhou, Guangdong, 510080, China

* Corresponding author: hlz6371@jnu.edu.cn, hewling@mail.sysu.edu.cn

^#^ The authors contributed equally to this work.


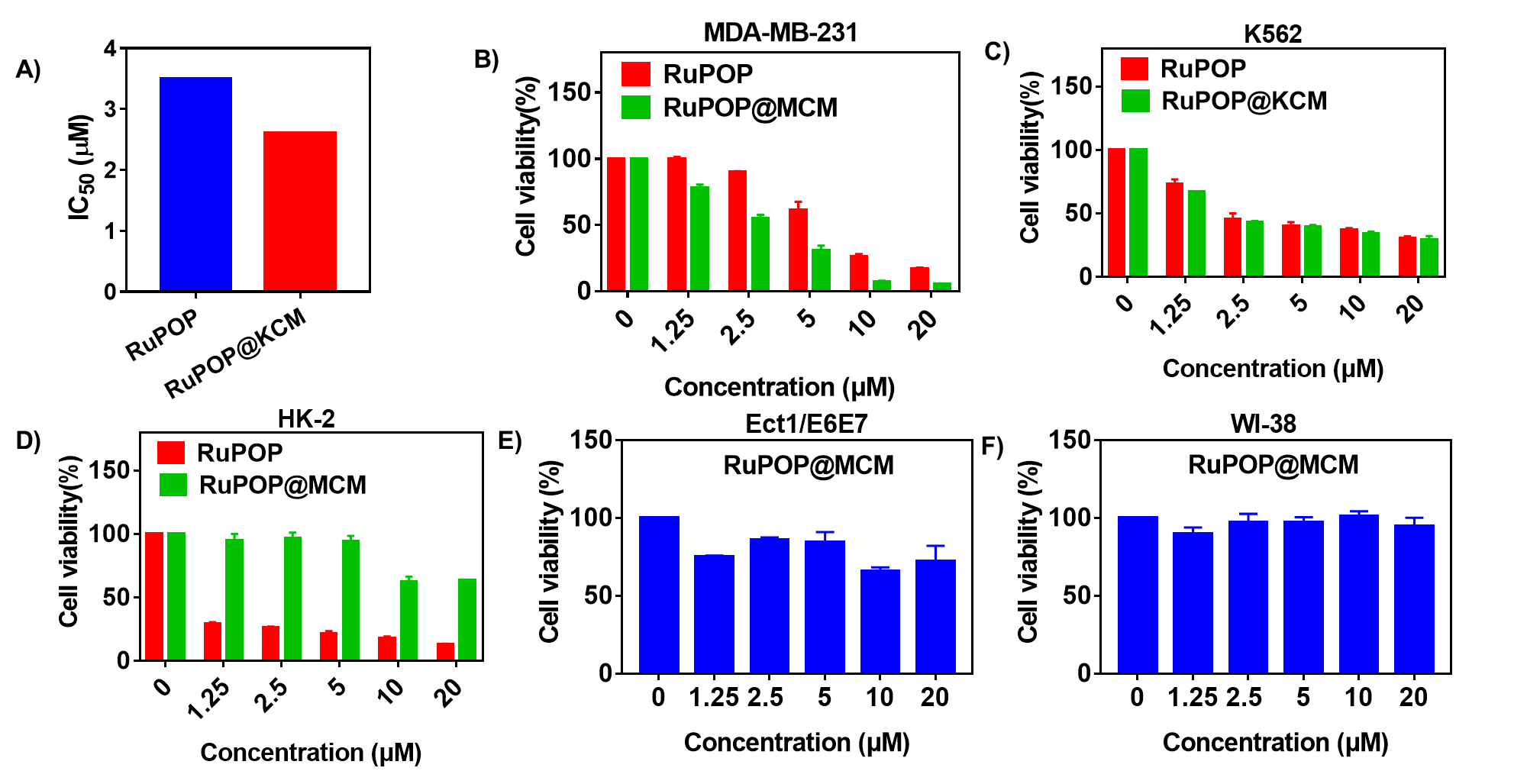


**Figure S1. RuPOP@CM exhibits excellent anti-tumor ability *in vitro*.** A) IC_50_ of K562 cells treated with different concentrations of RuPOP and RuPOP@KCM for 72 h. B) Cell viability of RuPOP@MCM and RuPOP treatment on MDA-MB-231 cells. C) Cell viability of RuPOP@KCM and RuPOP treatment on K562 cells K562 cells. Cell viability of RuPOP@MCM and RuPOP treatment on D) HK-2 cells, E) Ect1/E6E7 cells and F) WI-38 cells.


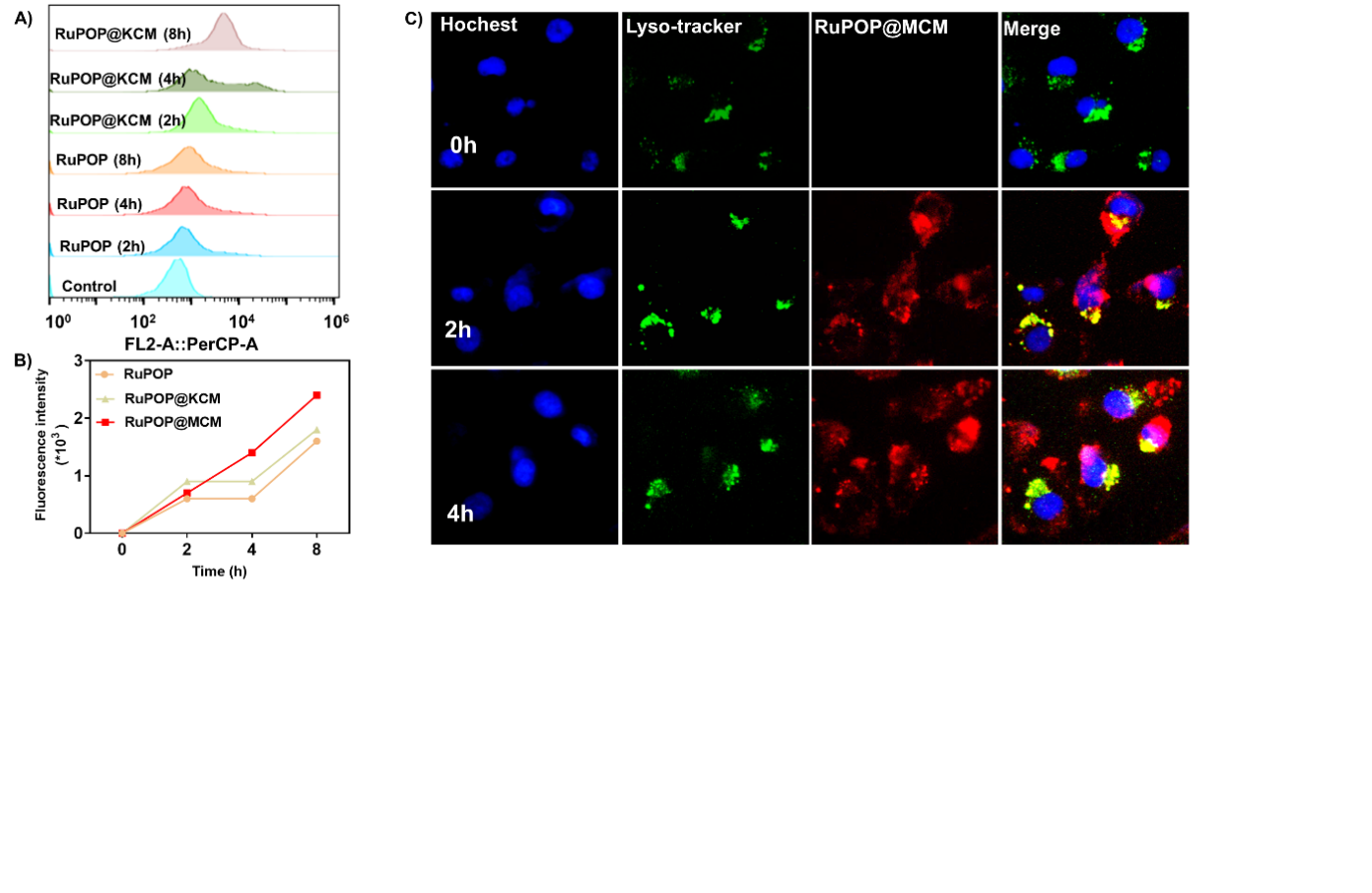


**Figure S2. Cellular uptake of RuPOP and KCM@RuPOP in MDA-MB-231 cells at different time.** A) Cellular uptake of RuPOP and RuPOP@KCM on MDA-MB-231 cells at different times. B) Cellular uptake of RuPOP@MCM, RuPOP and RuPOP@KCM in MDA-MB-231 cells by determination the fluorescence intensity of RuPOP. C) Translocation of RuPOP@MCM in MDA-MB-231 cells.


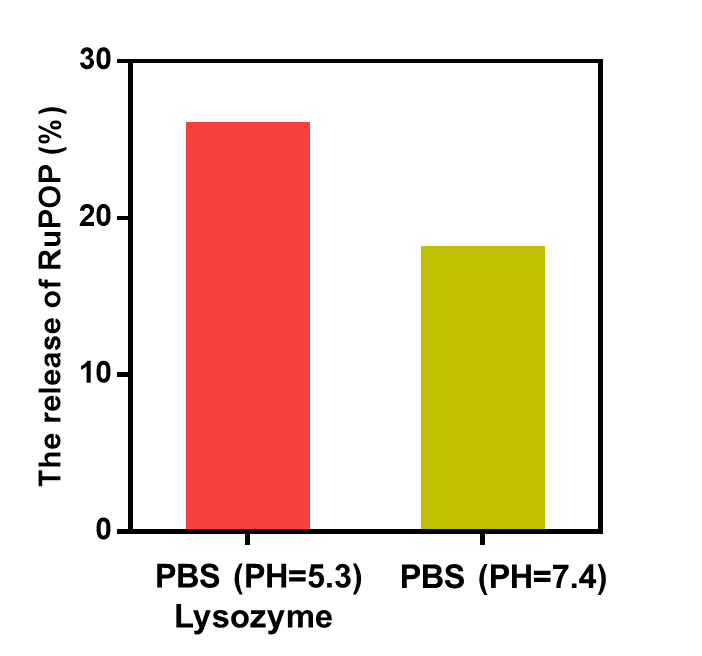


**Figure S3. Release concentration of RuPOP after incubation with PBS at pH 7.4 and PBS at pH 5.3 with lysozyme for 12 h.**

**Figure S4.** **IL-6 in serum from mice after being treated with RuPOP, RuPOP@MCM, RuPOP@KCM and LPS for 72 h respectively (*n* = 3 mice per group).**


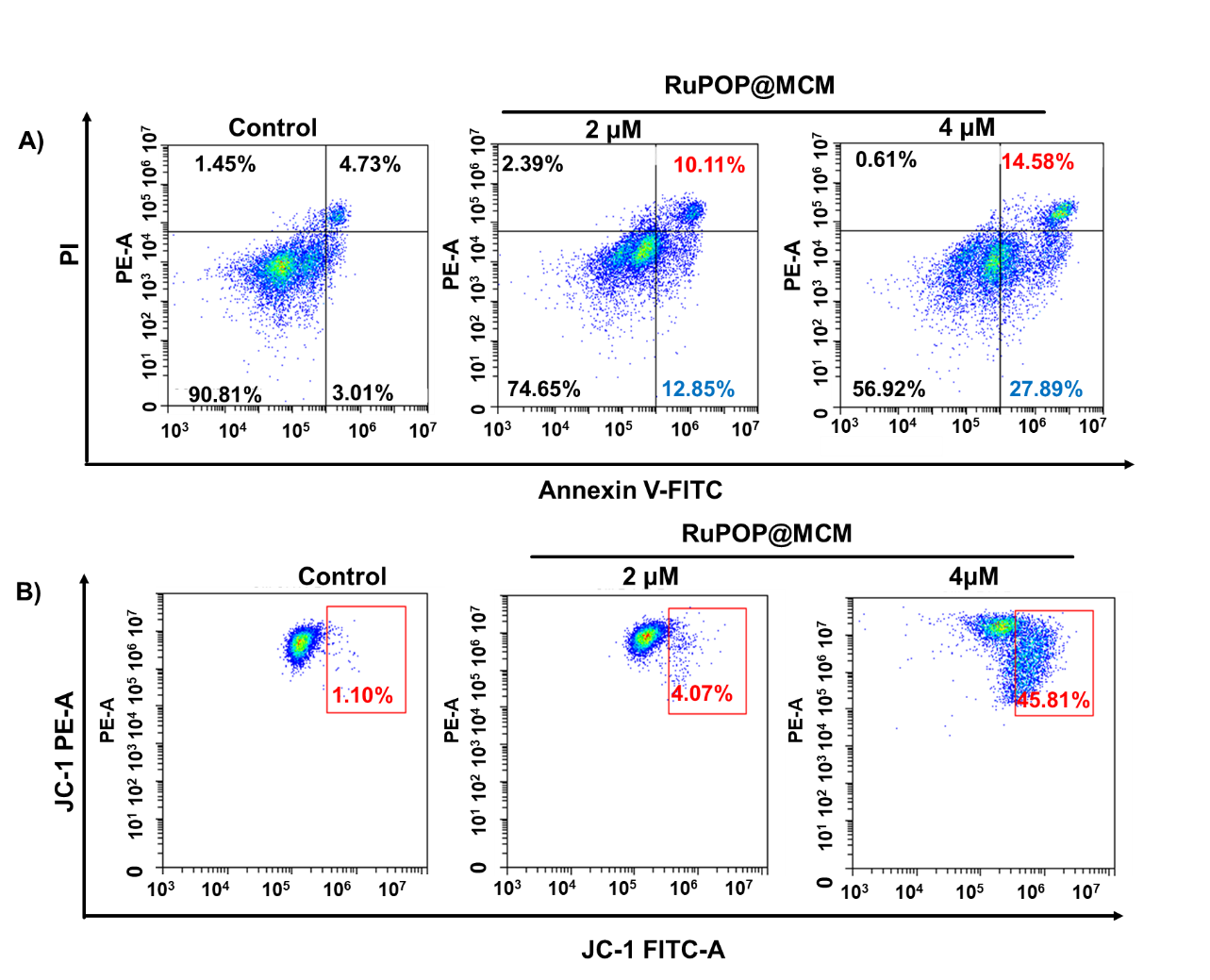


**Figure S5. Anticancer mechanism of RuPOP@CM *in vitro*.** A) Cell apoptosis induced by RuPOP@MCM. B) Mitochondrial membrane potential in MDA-MB-231 cells treated with RuPOP@MCM.

**
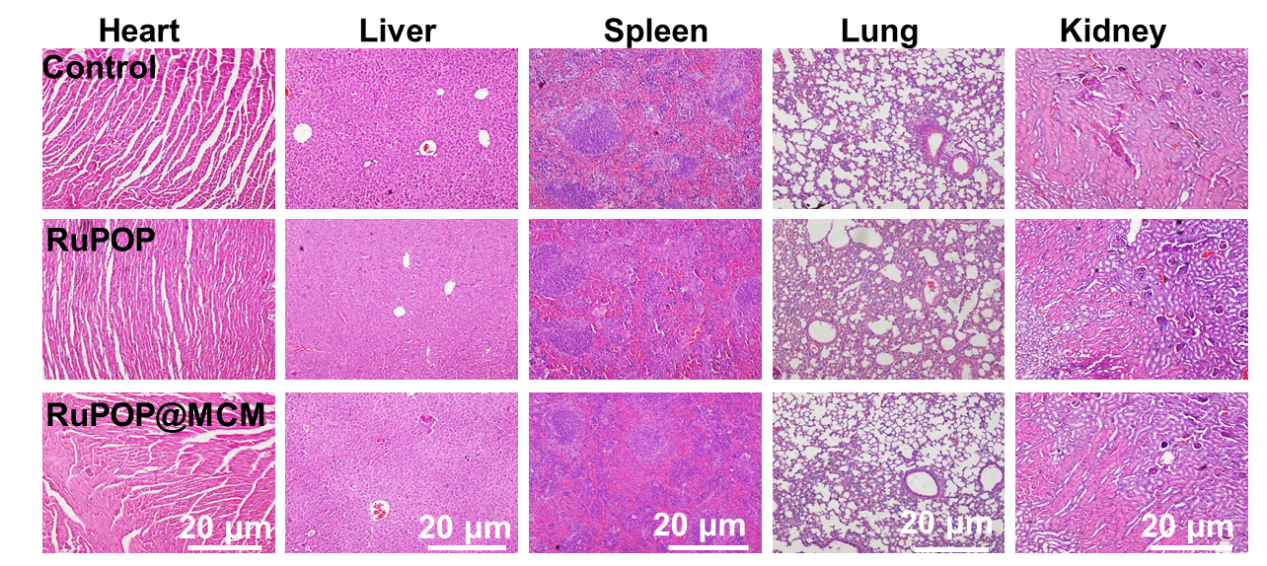
**

**Figure S6. H&E staining image of main organs from different groups.** Mice were treated with saline, RuPOP and RuPOP@MCM (1 mg/kg) through intravenous injection for 26 days.

**Table S1. Pharmacokinetic parameters of RuPOP**

| Parameter | Unit | Value |
| --- | --- | --- |
| K10 | 1/h | 1.25 |
| K12 | 1/h | 0.78 |
| K21 | 1/h | 0.17 |
| t1/2α | h | 0.33 |
| t1/2β | h | 6.97 |
| C0 | ng/ml | 4751.73 |
| V | (ng)/(ng/ml) | 0.08 |
| CL | (ng)/(ng/ml)/h | 0.10 |
| V2 | (ng)/(ng/ml) | 0.39 |
| CL2 | (ng)/(ng/ml)/h | 0.07 |
| AUC 0-t | ng/ml*h | 3322.56 |
| AUC 0-inf | ng/ml*h | 3812.56 |
| AUMC | ng/ml*h^2 | 17302.25 |
| MRT | h | 4.54 |
| Vss | ng/(ng/ml) | 0.48 |

**Table S2. Pharmacokinetic parameters of RuPOP@MCM**

| Parameter | Unit | Value |
| --- | --- | --- |
| K10 | 1/h | 0.49 |
| K12 | 1/h | 0.91 |
| K21 | 1/h | 0.54 |
| t1/2α | h | 0.39 |
| t1/2β | h | 4.74 |
| C0 | ng/ml | 7188.2 |
| V | (ng)/(ng/ml) | 0.06 |
| CL | (ng)/(ng/ml)/h | 0.03 |
| V2 | (ng)/(ng/ml) | 0.09 |
| CL2 | (ng)/(ng/ml)/h | 0.05 |
| AUC 0-t | ng/ml*h | 14456.34 |
| AUC 0-inf | ng/ml*h | 14808.18 |
| AUMC | ng/ml*h^2 | 82062.27 |
| MRT | h | 5.54 |
| Vss | ng/(ng/ml) | 0.15 |

**Table S3. Pharmacokinetic parameters of RuPOP@KCM**

| Parameter | Unit | Value |
| --- | --- | --- |
| K10 | 1/h | 0.67 |
| K12 | 1/h | 0.25 |
| K21 | 1/h | 0.36 |
| t1/2α | h | 0.66 |
| t1/2β | h | 3.03 |
| C0 | ng/ml | 1558.84 |
| V | (ng)/(ng/ml) | 0.26 |
| CL | (ng)/(ng/ml)/h | 0.17 |
| V2 | (ng)/(ng/ml) | 0.18 |
| CL2 | (ng)/(ng/ml)/h | 0.06 |
| AUC 0-t | ng/ml*h | 2271.50 |
| AUC 0-inf | ng/ml*h | 2341.64 |
| AUMC | ng/ml*h^2 | 5962.23 |
| MRT | h | 2.55 |
| Vss | ng/(ng/ml) | 0.43 |
